# Novel Two-Chamber Method for High-Precision TCR Determination of Current Shunts—Part II

**DOI:** 10.3390/s25216513

**Published:** 2025-10-22

**Authors:** Petar Mostarac, Roman Malarić, Hrvoje Hegeduš, Alan Šala

**Affiliations:** Faculty of Electrical Engineering and Computing, University of Zagreb, 10000 Zagreb, Croatia

**Keywords:** temperature coefficient of resistance (TCR), current shunts, measurement uncertainty of current shunts

## Abstract

This paper presents the experimental implementation and validation of the two-chamber method presented in Part I for the high-precision determination of the temperature coefficient of resistance (TCR) of current shunts. The two-chamber approach enables improved thermal isolation and independent temperature control of the reference and test shunts, which significantly reduces the measurement uncertainty. In this part, the complete experimental setup is described, including the thermoelectric temperature control, the current generation and the data acquisition system with synchronized high-resolution digital multimeters (DMMs). The experimental measurements were carried out for different resistance ratios ranging from 0.1 to 10. The results confirm the theoretical predictions and the uncertainty analysis from Part I. The influences of the stability of the current source, the temperature uniformity and the synchronization accuracy on the measurement results are evaluated. The two-chamber method shows high repeatability, ease of use and suitability for laboratory and interlaboratory tests, and thus represents a robust alternative to classical TCR determination methods.

## 1. Introduction

Accurate knowledge and management of the temperature coefficient of resistance (TCR) is critical to optimizing performance and ensuring the functional accuracy of electronic devices and high-precision measuring instruments. Even small changes in resistance caused by temperature fluctuations can lead to significant measurement errors, especially in critical applications such as meters, power distribution and battery testing. Therefore, understanding and accurately measuring the TCR of components such as current shunts is crucial for reliable system performance and minimizing measurement uncertainty [1,2,3].

The creation and characterization of new AC current shunts is an activity that our group of researchers has been engaged in scientific research about [4,5,6]. To overcome the challenges in the accurate determination of TCR, especially for precision current shunts, a novel two-chamber method was proposed and theoretically analyzed in Part I of this paper [6]. In Part I, the proposed method was described in detail, the influence of the individual parameters on the measurement uncertainty of the determined TCR was thoroughly analyzed and compared with the classical approach. It was shown that the new method offers significant advantages, including an improved stability of the reference shunt and a reduction in the overall measurement uncertainty, leading to a more accurate TCR determination. In addition, this method is applicable to a wide range of resistance ratios, especially from 0.1 to 10, is as easy to use as the classical method, offers a compact measurement setup and enables on-site measurements.

The novel two-chamber method was proposed and theoretically analyzed in Part I. It is designed for high-precision determination of the Temperature Coefficient of Resistance (TCR) of current shunts. The system uses two precision temperature chambers. Chamber 1 (Reference) maintains a constant reference temperature, usually $T_{0}=23\;{}^{\circ}C$. This chamber contains a calibrated reference shunt ($R_{1}$). Chamber 2 (Test) houses the device under test (DUT) shunt ($R_{t}$). Its temperature ($T_{t}$) varies within the temperature range of interest. The setup also includes a current source that excites both shunts with the same current (I), and high-precision digital multimeters (DMMs) for simultaneous measurement of the voltage drops across the shunts ($U_{1}$ and $U_{t}$). The core benefit of the two-chamber method is that it provides improved thermal isolation and independent temperature control of the reference and test shunts, significantly reducing measurement uncertainty compared to the classical approach. The TCR determination relies on indirect measurement of the resistance $R_{t}$. Instead of measuring $R_{t}$ directly, the current I is first calculated as the ratio of the voltage $U_{1}$ across the reference shunt to its known resistance $R_{1}$ using the expression $I=U_{1}/R_{1}$. Subsequently, the resistance of the test shunt ($R_{t}$) is calculated using the measured voltage $U_{t}$ and the calculated current I: $R_{t}=U_{t}/I$, or $R_{t}=(U_{t}/U_{1})R_{1}$. By keeping the temperature of the reference shunt ($R_{1}$) constant, the method ensures stable determination of the current flowing through the shunts.

Building on the theoretical framework and uncertainty analysis presented in Part I, this second article, Part II, focuses on the experimental implementation and comprehensive validation of the novel two-chamber method. The main objective of Part II is to demonstrate the practical application of the measurement method and equipment to calculate the TCR for resistive current shunts at different temperatures.

This includes the two-chamber system based on solid-state thermoelectric pumps, the measurement system with high-resolution digitizers (DMMs) and the graphical interface for data input, analysis and visualization. In addition, the results of experimental measurements for three different resistance ratios (0.1, 1 and 10) are presented to empirically test the system and the proposed method.

Furthermore, the experimental results are compared with the predicted uncertainty bounds derived from the theoretical analysis in Part I to verify the effectiveness of the method in practice. Important experimental factors such as the accuracy of the temperature measurements in the chambers, the influence of the stability of the current source and the effects of the synchronization of the two DMMs are discussed. In addition, the repeatability of the obtained TCR results is analyzed, which further underlines the reliability of the method. Finally, the practical aspects of the method, such as its time efficiency, complexity and ease of use, are highlighted and its potential for automation and customization for intercomparisons is discussed.

In precision resistance metrology, direct current comparator (DCC) bridges enable accurate comparisons between resistors with values differing by several decades. Ratios of 0.01 and 100 (i.e., 1:100 and 100:1) can be achieved by selecting appropriate current-comparator winding ratios, allowing a low-value shunt to be compared directly to a much higher-value standard, and vice versa, with very low uncertainty [7]. Because measurement with a DCC is based on current ratio rather than the absolute voltage across the standards, DCC techniques are particularly well suited to small-resistance artifacts such as current shunts, providing stable, low-burden comparisons within a traceable framework used by national metrology institutes.

This Part II therefore serves as decisive experimental evidence confirming the theoretical advances and advantages of the novel two-chamber method presented in Part I for high-precision TCR determination of current shunts.

## 2. Experimental Setup

The experimental setup consisted of two thermoelectric chambers for temperature control, a customized relay switching unit, a precision current source and two high-precision digital multimeters, all coordinated by a higher-level LabVIEW application, with the CR1000X data logger (Campbell Scientific, Inc., Logan, UT, USA) providing temperature control and stability monitoring.

### 2.1. The Two-Chamber System (Mechanical, Thermal and Electrical Aspects)

**Figure 1:**
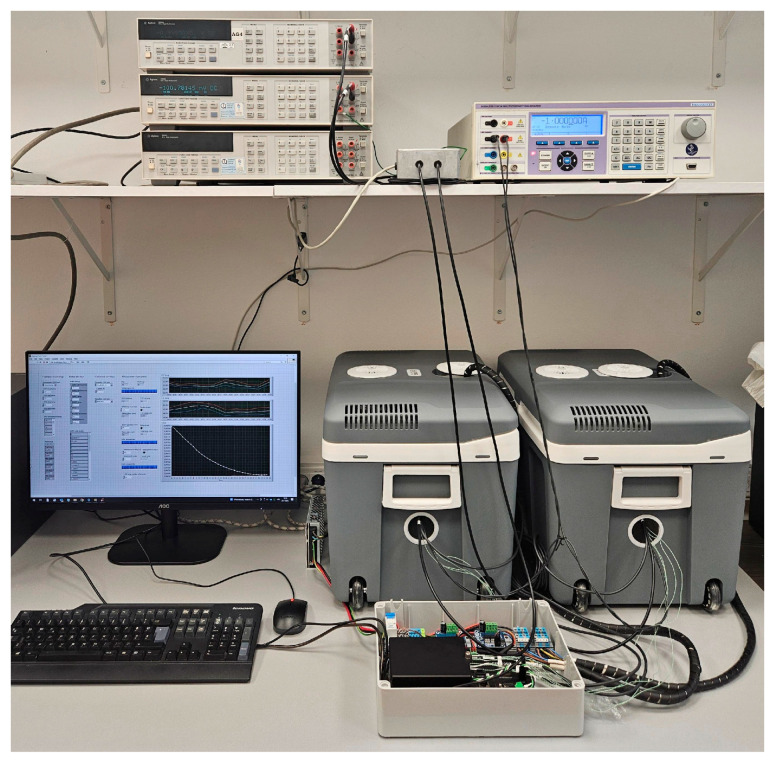
Experimental setup.

For the purposes of this study, two thermoelectric chambers based on Peltier elements were used (Figure 1). These chambers, consisting of commercially available 40-L coolers, were mechanically and electrically modified to allow precise regulation of the internal temperature. A Campbell Scientific CR1000X data logger [8] was used to measure temperature and control the heating and cooling process.

The original insulated enclosures of the coolers were retained to provide thermal insulation, while internal modifications allowed for stable and uniform temperature conditions. Each chamber contains a centrally mounted support for the device under test, with free air circulation maintained by an axial fan. The fans are powered by the switched 12 V outputs of the CR1000X and operate during regulation to homogenize the chamber atmosphere and adequately cool the heat sinks of the thermoelectric modules. The electrical and sensor connections are routed through sealed feedthroughs in the chamber walls to minimize leakage and thermal interference.

Temperature regulation is achieved by the Peltier modules integrated into the walls of the cooler. Each module is powered by a regulated 12 V DC supply via a special H-bridge driver, which enables current reversal and thus allows both heating and cooling. With this configuration, the chambers can be stabilized within an operating range of approximately 10 °C to 45 °C.

**Figure 2:**
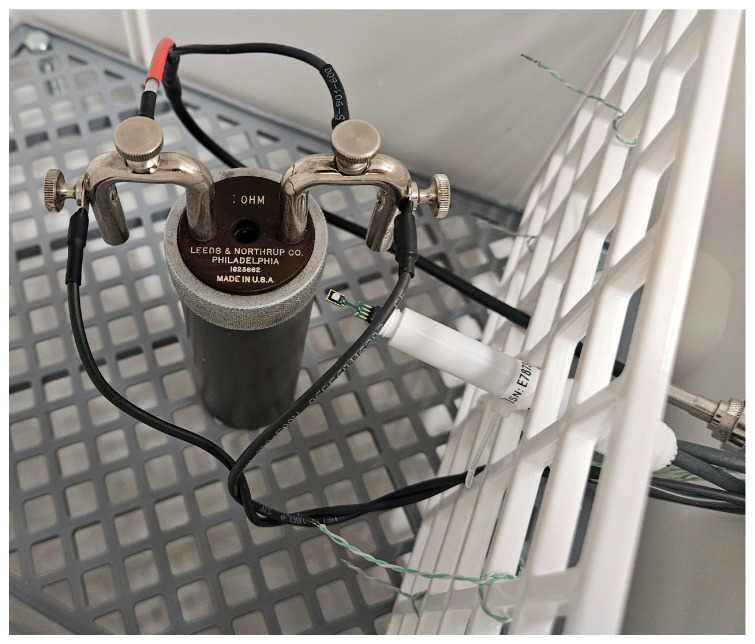
Inside the thermoelectric chamber, shunt with temperature probe and thermocouples.

The air temperature is monitored by a combination of a HygroVUE5 sensor (Campbell Scientific, Inc., Logan, UT, USA) and four thermocouples in each chamber (Figure 2). The HygroVUE5 [9] is positioned in the center of the chamber near the device under test and provides a calibrated and accurate reference measurement. The thermocouples are type K, with the junctions welded to a single point of very low thermal mass. This design minimizes thermal inertia and reduces response time, allowing the thermocouples to detect rapid fluctuations in air temperature. The sensors are arranged in a two-by-two matrix around the device under test, with two at one-third and two at two-thirds of the device height and distributed laterally on the left and right sides of the chamber. This configuration enables the detection of vertical and horizontal gradients while providing responsive data for control.

The thermocouples are sampled every second, while the HygroVUE5 sensor is polled every five seconds via the SDI-12 interface. A signal fusion approach was implemented to utilize the complementary properties of the two sensor types. The average temperature of the thermocouple is processed through a high-pass filter with a time constant of 180 s, which removes the slow drift while retaining the fast fluctuations. In parallel, the HygroVUE5 measurement is processed through a low-pass filter with the same time constant, which suppresses noise and provides a stable long-term reference. The process temperature delivered to the controller is the sum of the high-pass-filtered thermocouple signal and the low-pass-filtered HygroVUE5 signal. This complementary filtering strategy ensures both the accuracy and dynamic responsiveness of the feedback signal.

The CR1000X runs a digital PID algorithm with a control speed of one to two Hertz. The error signal is calculated as the difference between the temperature setpoint and the process temperature. The PID output is a signed variable, where the sign defines the polarity of the H-bridge (heating or cooling mode) and the magnitude defines the PWM duty cycle applied to the thermoelectric module. The fans are controlled simultaneously to ensure a uniform atmosphere in the chamber and stable operation of the thermoelectric elements.

The temperature data from both chambers is stored on the CR1000X at one-minute intervals. Each recorded value represents the arithmetic mean of the one-second temperature samples taken during the previous minute. This averaging reduces the influence of short-term fluctuations and noise and provides a representative measurement of chamber air temperature for stability assessment.

### 2.2. Description of Relay Box

To enable controlled switching of the two Agilent 3458A (Agilent Technologies, Inc., Santa Clara, CA, USA) [10] digital multimeters to the resistors under test, a customized relay switching unit was developed. The unit uses two Panasonic TXS2-L-4 single coil (Panasonic, Osaka, Japan), latching signal relays (rated coil voltage 4.5 V) selected for their low contact resistance and low thermal electromotive force, making them suitable for precision resistance measurements.

The actuation of the relays is controlled by a special Arduino Nano PCB. The controller generates bipolar control signals via two H-bridge stages that apply a positive polarity for the set operation and a negative polarity for the reset operation. The duration of the control pulses is 2 ms, a value that has been experimentally determined to ensure reliable latching while minimizing coil heating and associated thermal gradients. The Arduino is powered via a standard USB connector that supplies 5 V. The same 5 V rail is also used to control the relays. The low voltage drop across the H-bridge transistors results in an effective coil voltage that is very close to the nominal 4.5 V and ensures optimal operating conditions.

To further suppress thermal and electromagnetic influences, the relays have been mounted on a precision-machined copper block with two recesses into which the relay housings fit exactly. This design ensures that both relays remain at the same temperature during operation. The copper block is housed in a small aluminum housing (90 × 36 × 30 mm), which in turn is housed in a larger aluminum housing (105 × 90 × 55 mm). This double-shielded design ensures both thermal equalization and effective electromagnetic shielding of the switching module.

RG59 coaxial cables are used for the signal connections to the relay box to minimize noise pick-up and thermoelectric effects. Two RG59 cables lead the voltage measurement lines from the resistors $R_{1}$ and $R_{2}$ into the box and are connected directly to the relay connections to avoid unnecessary transitions. An RG59 cable is routed from each relay directly to the input terminals of each voltmeter. Relay 1 is configured so that in the SET state, resistor $R_{1}$ is connected to voltmeter $V_{1}$, while in the RESET state, resistor $R_{2}$ is connected to $V_{1}$. Relay 2 operates in the same way, with $R_{1}$ connected to $V_{2}$ in the SET state and $R_{2}$ connected to $V_{2}$ in the RESET state.

This configuration allows a controlled exchange of the voltmeter-resistor assignments without changing the external wiring. In combination with the current polarity reversal provided by the Transmille source [11], the system ensures that each resistance is measured by both voltmeters at both current polarities. This process effectively eliminates systematic effects such as thermal electromotive forces, device offsets and channel-specific errors, supporting highly accurate resistance measurement.

### 2.3. Description of the Current Source, DMMs, Switching and Synchronization

The resistors to be tested were excited by a Transmille precision current source, which supplied a stable and traceable current. Voltage and resistance measurements were made using two Agilent 3458A digital multimeters operated in four-wire mode. The custom relay box allowed controlled replacement of the voltmeter resistor connections as described above.

The higher-level LabVIEW [12] application controlled the Transmille source, the relay box and the two multimeters. All devices were synchronized via software triggers, ensuring that each resistance measurement was accurately correlated to the stabilized chamber temperature recorded by the CR1000X. All recorded data was time-stamped and stored for later analysis.

### 2.4. Temperature Control System and Its Calibration

The CR1000X data logger sampled the thermocouples every second and the HygroVUE5 probes every five seconds. The complementary filter approach (high-pass-filtered thermocouples, low-pass-filtered HygroVUE5) gave the process temperature used for PID control. The temperature measurement system was calibrated by comparing the HygroVUE5 probes with a reference thermometer, while the thermocouples were verified by cross-comparison within the chambers. Uniformity tests confirmed that the vertical and lateral temperature gradients within the chambers remained below 0.1 °C under stable conditions.

The stability criterion was defined as a deviation of less than ±0.01 °C from the set point for at least 20 consecutive minutes. Once stability was achieved, an additional 30-min waiting period was introduced to ensure thermal equilibration of the device under test with the chamber air before electrical measurements were performed.

### 2.5. Measurement Procedure

The temperature setpoint for each chamber was determined by the LabVIEW application communicating with the CR1000X. The chambers were stabilized using the PID control system described above. The stability of the chambers was checked by evaluating the one-minute average temperatures stored on the data logger. If the stability criterion was met, a waiting period of 30 min was observed to ensure equilibration of the device under test. The resistance measurements of the shunts were then performed at the stabilized temperature. The procedure was repeated for each programmed setpoint in the range of 10–45 °C.

**Figure 3:**
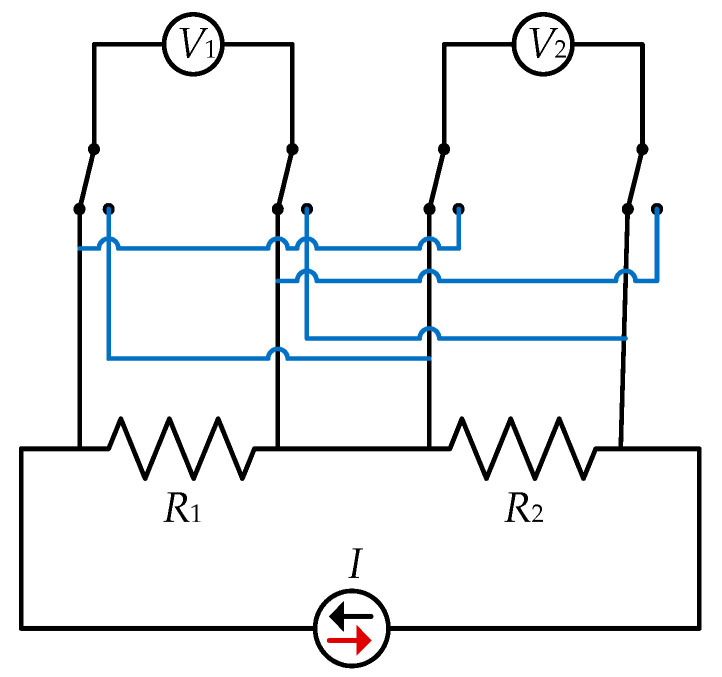
The electrical schematic of the measurement setup.

In the dual voltmeter, dual chamber configuration, the Transmille calibrator provided current excitations across two resistors, $R_{1}$ and $R_{2}$, while voltage drops were measured simultaneously using two 3458A digital multimeters $V_{1}$ and $V_{2}$ (Figure 3). The relay box allowed the channels to be swapped so that both voltmeters measured each resistor in turn. In addition, the current polarity was reversed to compensate for thermal EMF effects.

A complete measurement sequence consisted of four steps:

$$\mathrm{step}\;1:\;\mathrm{positive}\;\mathrm{current},\;V_{1}\;measureson\;R_{1}\;and{\;V}_{2}\;measureson\;{\;R}_{2}\;\mathrm{step}\;2:\;\mathrm{positive}\;\mathrm{current},\;V_{2}\;measureson\;R_{1}\;and{\;V}_{1}\;measureson\;{\;R}_{2}\;\mathrm{step}\;3:\;\mathrm{negative}\;\mathrm{current}:\;V_{1}\;measureson\;R_{1}\;and{\;V}_{2}\;measureson\;{\;R}_{2}\;\mathrm{step}\;4:\;\mathrm{negative}\;\mathrm{current}:\;V_{2}\;measureson\;R_{1}\;and{\;V}_{1}\;measureson\;{\;R}_{2}$$

At each step, ten measured values per voltmeter were recorded with an integration time of 10 NPLC. Each step was followed by a pause of 120 s to allow transient effects to subside. By averaging the results over all four steps, systematic errors were eliminated so that high-precision resistance values could be determined under stable thermal conditions.

In addition to the configuration with two chambers, a classic four-wire method was also used. In this approach, a single resistor was placed in a temperature chamber and excited by the Transmille precision current source. The voltage drop across the resistor was measured with a single 3458A multimeter in four-wire mode, using separate current and test leads to eliminate the influence of lead resistance.

This configuration simplified the measurement chain as no relay box was required and systematic errors due to lead resistance were inherently suppressed by the 4 wire connection. However, as only one resistor could be measured at a time, the measurement throughput was lower compared to the two-chambers method. The same stability criteria, timing and averaging rules applied: ten measurements with 10 NPLC integrations per measurement point, separated by defined waiting times after thermal stabilization of the chamber.

## 3. Results

Measurements were carried out for resistance ratios: 0.1, 1, 10. The ratio is defined by the expression (1). The maximum allowable shunt temperature was considered so that a higher nominal resistance value connected in series with a lower resistance would not cause the larger resistance to overheat and damage the device, which would lead to incorrect and distorted measurement results. The results are presented in three subsections, with one subsection reserved for each ratio to clearly and unambiguously present the measurement results. The results shown are the measured resistance $R_{2}$ with the two-chamber method and the measured $R_{2}$ with the one-chamber method (four-wire resistance measurement). In addition, the TCRr (relative TCR) calculated from individual measurements with both methods is presented to illustrate the applicability of the two-chamber method.

The resistance ratio is defined as:

(1) $$r=\frac{R_{1}}{R_{0}}.$$

The $R_{0}$ is the resistance of $R_{2}$ at reference temperature, the nominal values are used when calculating the ratio. This section also shows the stability of the temperatures in the chambers for both measurements. For two-chamber measurements, the results of temperature stability in both chambers are shown, and for single-chamber systems, the results of temperature stability in the chamber during a four-wire resistance measurement with a DMM are shown.

The nominal values of the shunt resistors are used to present the results. The reference resistance ($R_{1}$) in chamber 1 has a nominal resistance of 1 Ω. And the resistors in chamber 2 ($R_{2}$), the resistors whose TCR is determined, have values of 10, 1 and 0.1 Ω respectively. In this way you obtain ratios r of 0.1, 1 and 10 according to (1). Throughout the chapter, nominal values are used to reduce the notation of decimal numbers and to reduce the possibility of misinterpretation by introducing nominal currents for which the shunts are designed. All exact data can be found in Table 1.

The measurement uncertainty was determined according to the analysis described in the previous article (Part I) using the defined components B of the measurement uncertainty of the temperature sensor and component A of the measurement uncertainty obtained by measuring individual variables ($R_{0},\;T_{t},\;T_{0}$).

The measurement uncertainty was determined according to the instructions of the GUM [13] as the sum of the independent variables (the square root of the squares of A and the B component of the measurement uncertainty) and propagated through the sensitivity coefficients in the calculation of indirect measurements such as the determination of $R_{2}$ from the voltage ratio and the determination of TCR from $R_{2}$. The mathematical description can be found in Part I [6].

### 3.1. Resistance Ratio in the Chambers r = 0.1

**Figure 4:**
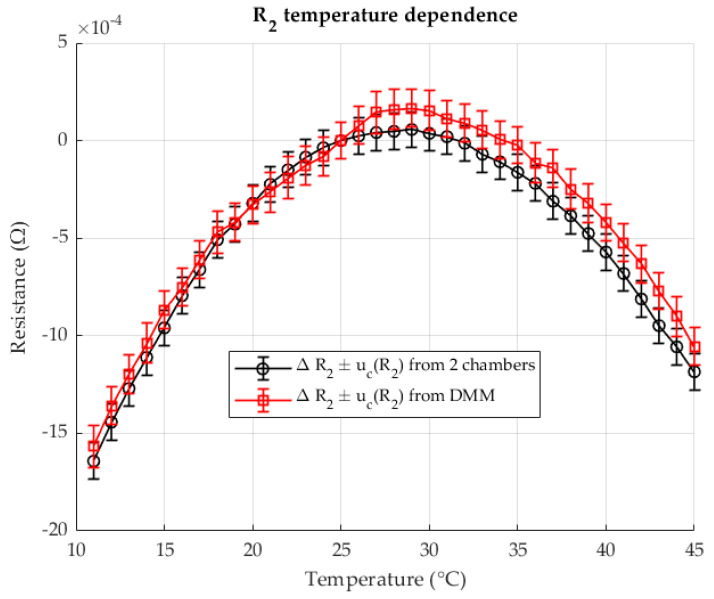
The temperature dependence of the resistance $R_{2}$, measured with two chambers (black) and comparison with the directly measured resistance (red). Resistance ratio *r* = 0.1 Ω/Ω.

Figure 4 shows the change in resistance $R_{2}$ in chamber 2 as a function of the change in temperature $T_{2}$. The results are shown for the two-chamber method and for the direct resistance measurement with a DMM (four-wire method). The resistances are given as differences (delta) in relation to the resistance at a reference temperature of 23 °C.

**Figure 5:**
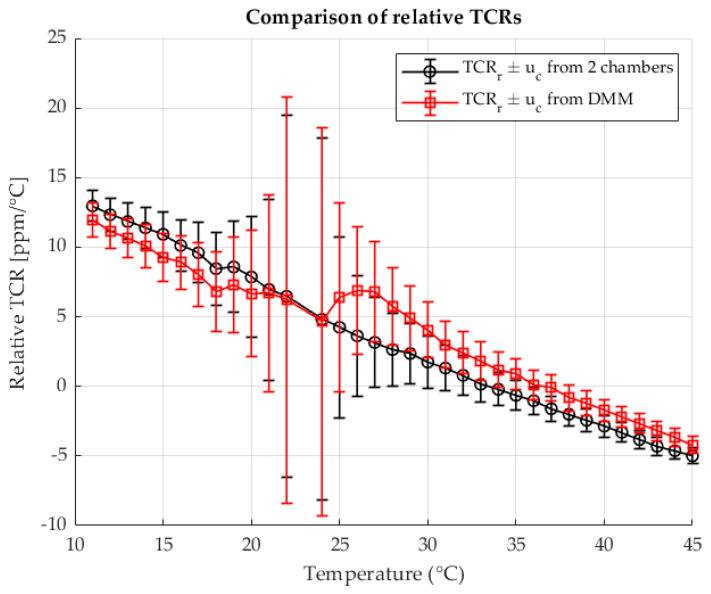
Relative TCRs calculated from $R_{2}$ for the two chambers method (black) and the four wire DMM measurement (red).

The following Figure 5 shows the calculated relative TCRs for each measured point for both methods. The results are shown as a function of the temperature in chamber 2. The results are shown with the associated measurement uncertainties.

When determining resistance, good agreement is seen between the results of both methods. Direct resistance measurement with a DMM is most efficient in this ratio r=0.1 Ω/Ω. The resistance value in chamber 2 is of an ideal value in relation to the measurement range of the DMM. Therefore, the TCR results are very similar and coincide. This confirms that the two-chamber method is as good as direct resistance measurement when the resistance is of an appropriate value that allows maximum use of the DMM’s precision within the measurement range. The analysis below will show that this is not the case for smaller values of $R_{2}$. In less favorable ratios, the two-chamber method will show significantly better results.

**Figure 6:**
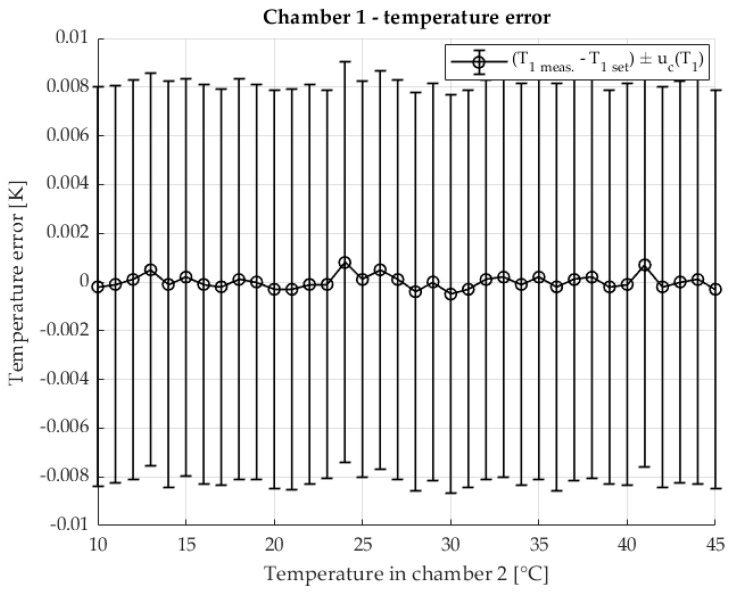
Temperature error of $T_{1}$ in chamber 1 in two chamber method. Set temperature is 23 °C.

The temperature stability in chamber 1 is shown in Figure 6 The error of the measured temperature inside the chamber in relation to the set temperature is shown. The measurement uncertainty of the temperature is also shown. The temperature range corresponds to the temperature range at which the TCR shunt $R_{2}$ was measured.

**Figure 7:**
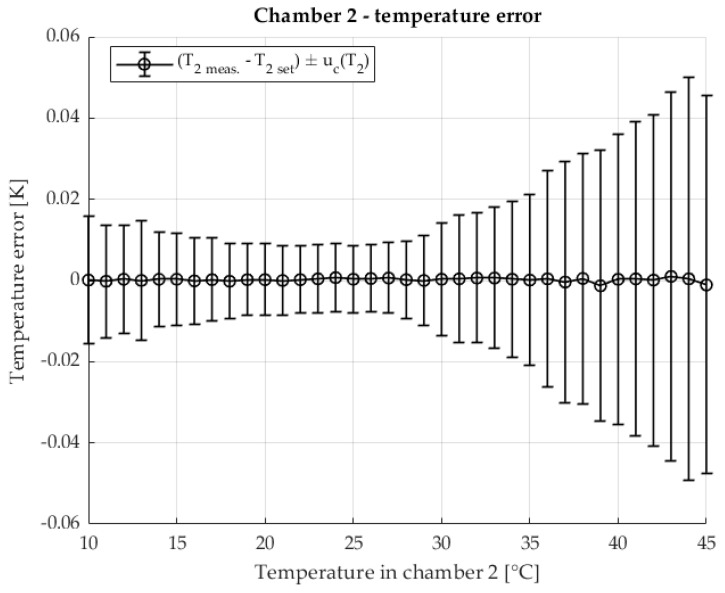
Temperature error of $T_{2}$ in chamber 2 in two chamber method.

The temperature stability in the second chamber containing the DUT is shown in Figure 7 The temperature of the second chamber is shown as an error relative to the set temperature. The results are shown with the associated measurement uncertainty.

The temperature in the first chamber is very stable. Chamber 1 is at a stable 23 °C throughout the measurement. Very stable temperature conditions are also ensured in chamber 2. At higher temperatures, an increase in measurement uncertainty is visible, where component A dominates due to the increase in the disturbance quantity (the laboratory is at a temperature of 23 °C) and the power of the heating element.

**Figure 8:**
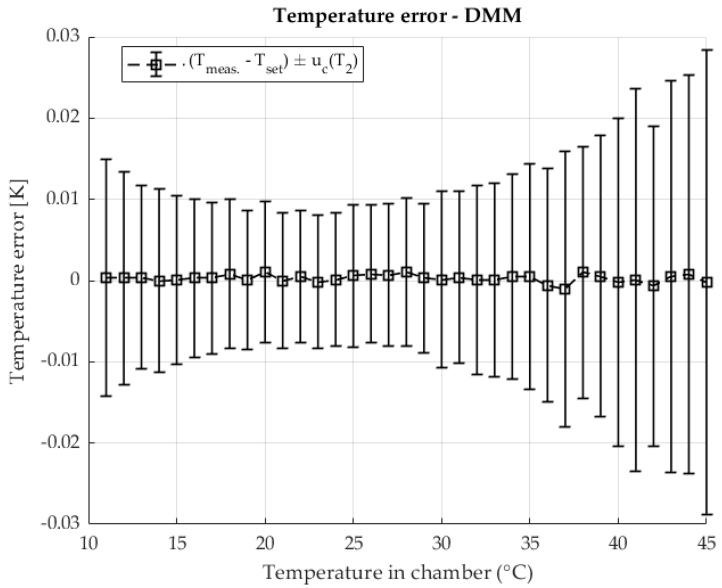
The temperature error in chamber during the resistance measurement with DMM.

The temperature stability when measuring shunt resistance with a DMM using the four-wire method is shown in Figure 8 The result shows errors relative to the set temperature along the areas defined by the measurement uncertainty of the temperature measurement.

Stable temperature conditions were ensured in the chamber used during the resistance measurement with the DMM. Similarly to the previous two-chamber method, in this method, at higher temperatures, an increase in measurement uncertainty is visible, where component A dominates due to the increase in the contribution of the disturbance quantity and the power of the heating element.

### 3.2. Resistance Ratio in the Chambers r = 1

**Figure 9:**
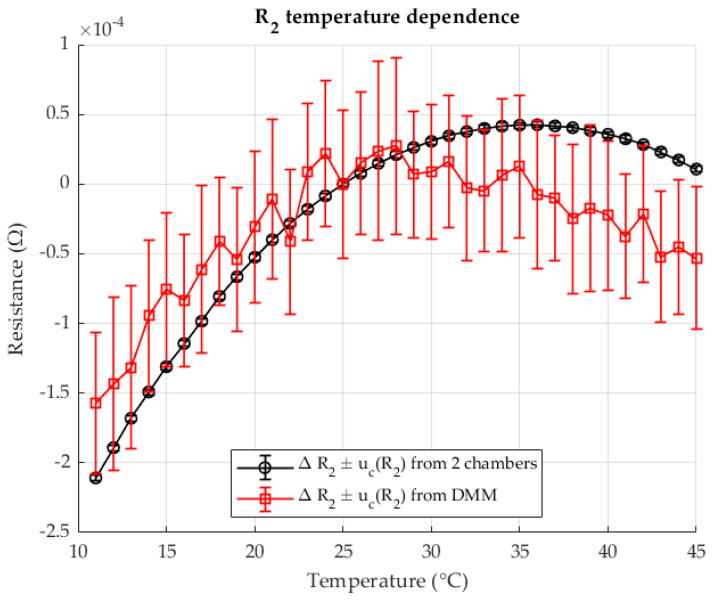
The temperature dependence of the resistance $R_{2}$, measured with two-chambers (black) and comparison with the directly measured resistance (red). Resistance ratio *r* = 0.1 Ω/Ω.

As shown in Figure 9, the resistance $R_{2}$ in chamber 2 varies with temperature. Results are presented for both the proposed two-chamber method and direct resistance measurement using a DMM (four-wire method). The resistances are expressed as deviations (Δ*R*) relative to the reference resistance at 23 °C.

**Figure 10:**
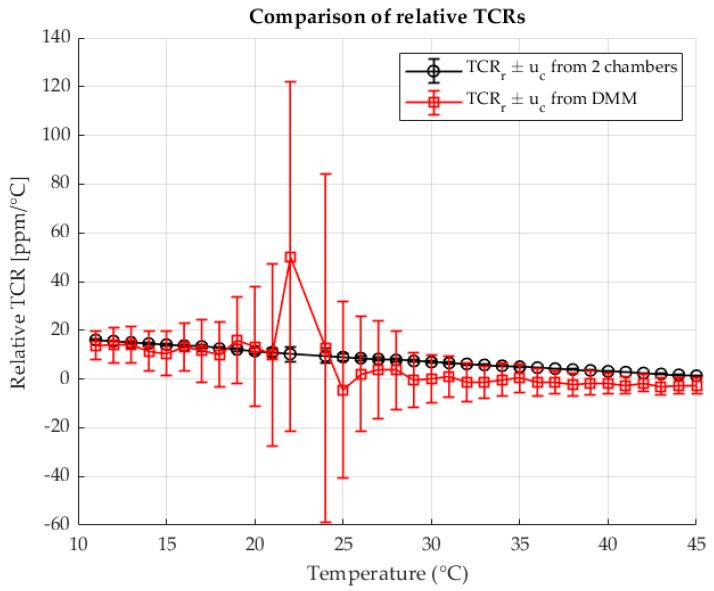
Relative TCRs calculated from $R_{2}$ for the two-chambers method (black) and the four wire DMM measurement (red). Resistance ratio *r* = 1 Ω/Ω.

Figure 10 presents the relative TCR values calculated at each measurement point for both methods. The results are plotted as a function of the shunt temperature, with the corresponding measurement uncertainties indicated.

Both methods agree well in their results. A certain deviation is visible at higher temperatures if you measure the resistance $R_{2}$ with a DMM. The results obtained with the two-chamber method show a smooth curve of the calculated resistance $R_{2}$ with a significantly lower measurement uncertainty. The TCR results obtained with the DMM measurement show a fluctuation of the results around the reference temperature. The results obtained with the two-chamber method show a more even curve of the TCR change with a significantly lower measurement uncertainty. The trends of the curves match.

**Figure 11:**
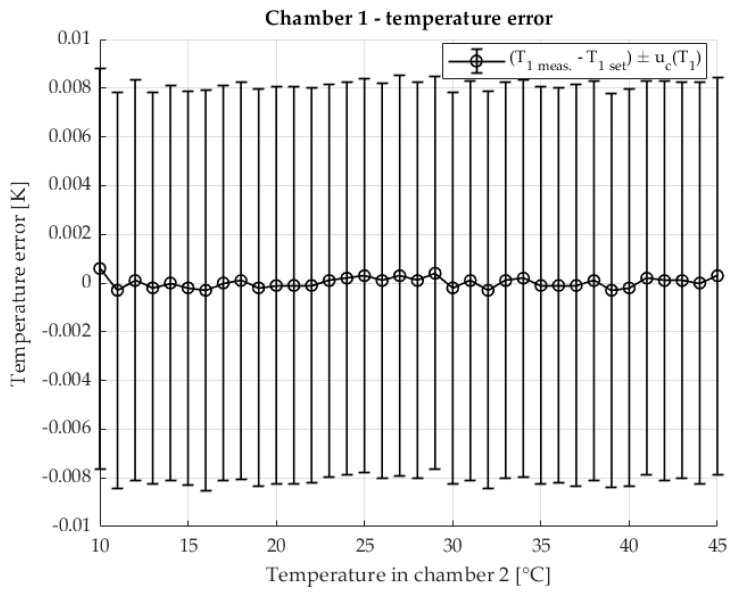
Temperature error of $T_{1}$ in chamber 1 with the two-chamber method. The set temperature is 23 °C. Resistance ratio *r* = 1 Ω/Ω.

Figure 11 shows the temperature stability of chamber 1. The deviation of the measured chamber temperature from the programmed setpoint is displayed together with the associated measurement uncertainty. The temperature range covered corresponds to that used for the TCR measurements of shunt $R_{2}$.

**Figure 12:**
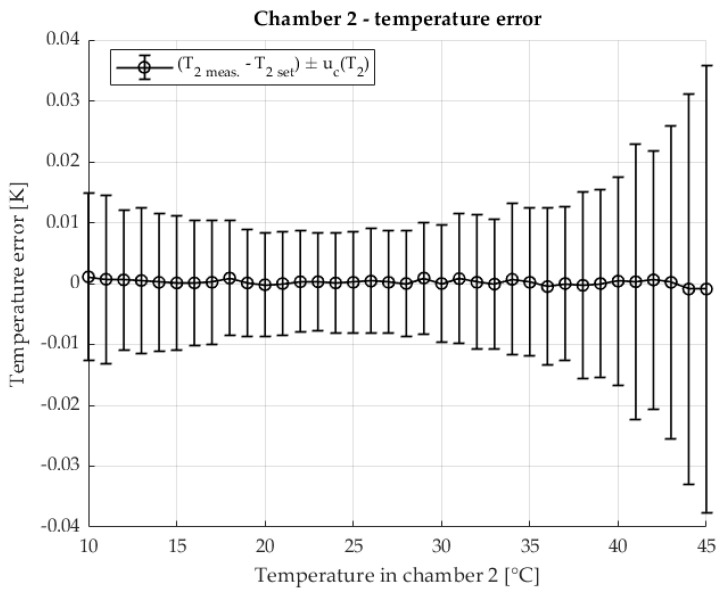
Temperature error of $T_{2}$ in chamber 2 with the two-chamber method. Resistance ratio *r* = 1 Ω/Ω.

The temperature stability of chamber 2, in which the device under test is located, is shown in Figure 12 The measured chamber temperature is shown as a deviation from the setpoint value with the corresponding measurement uncertainties.

**Figure 13:**
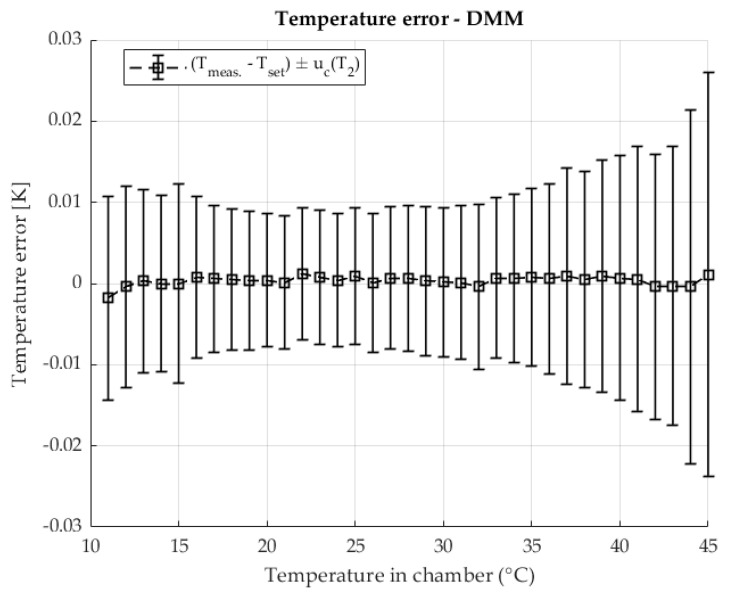
The temperature error in the chamber during the resistance measurement with the DMM. Resistance ratio *r* = 1 Ω/Ω

Figure 13 illustrates the temperature stability of resistance measurements made with a DMM using the four-wire method. The results show the deviation from the programmed setpoint with the uncertainty of the temperature measurement.

The temperatures are very stable with both methods. There is a visible increase in measurement uncertainty due to temperature fluctuations in the chambers when measurements are taken at higher and lower temperatures. At these extreme points, the limits of the heating and cooling systems and the influence of the laboratory temperature as a disturbance variable are pronounced.

### 3.3. Resistance Ratio in the Chambers r = 10

**Figure 14:**
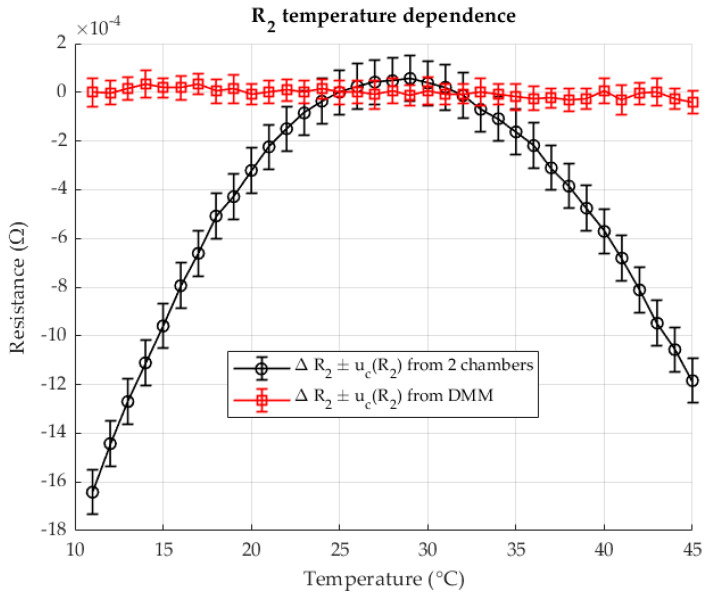
The temperature dependence of the resistance $R_{2}$, measured with two-chambers (black) and comparison with the directly measured resistance (red). Resistance ratio *r* = 1 Ω/Ω.

Figure 14 shows the change in resistance $R_{2}$ in chamber 2 as a function of temperature. The results are given both for the two-chamber method and for the direct resistance measurement with a DMM in four-wire configuration. All resistances are given as deviations (ΔR) relative to the reference resistance at 23 °C.

**Figure 15:**
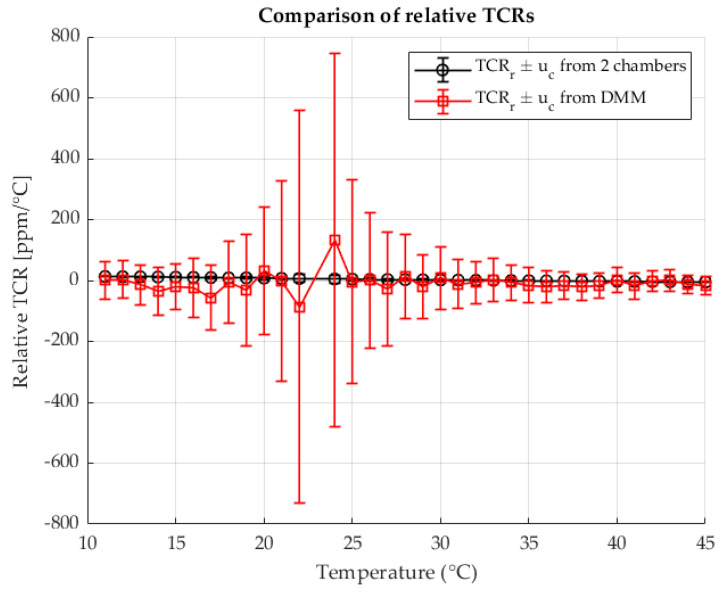
Relative TCRs calculated from $R_{2}$ for the two-chambers method (black) and the four wire DMM measurement (red). Resistance ratio *r* = 0.1 Ω/Ω.

Figure 15 shows the relative TCR values calculated for each measurement point using both methods. The values are plotted against the temperature of the shunt, with the associated measurement uncertainties indicated.

Extremely unfavorable results were obtained when measuring the resistance with a DMM. Due to the nominal resistance value $R_{2}$ = 0.1 Ω, the measuring range of the device, which is a hundred times greater than the nominal value of the measured resistance, is very poorly utilized. In addition, there is a drastic increase in measurement uncertainty. The insufficiently good results of the $R_{2}$ measurement also led to the calculation of a very inferior TCR with a large measurement uncertainty. However, the two-chamber method remains consistent with the previous results and the analyzes carried out previously and manages to measure the change in resistance with temperature and to determine a high-quality TCR based on this data even at this resistance ratio.

**Figure 16:**
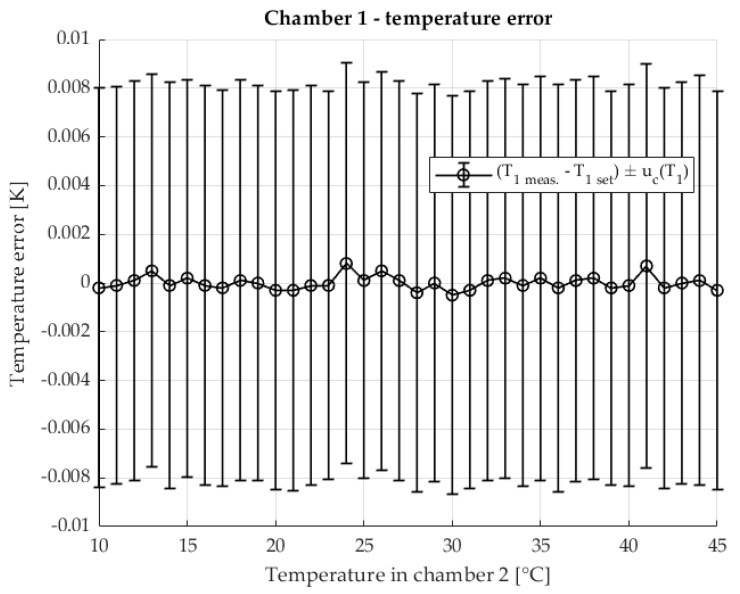
Temperature error of $T_{1}$ in chamber 1 in two-chamber method. The set temperature is 23 °C. Resistance ratio *r* = 0.1 Ω/Ω.

Figure 16 illustrates the temperature stability of chamber 1. The deviation of the measured chamber temperature from the programmed target value is shown together with the measurement uncertainty. The temperature range corresponds to the interval used for the TCR measurements of shunt $R_{2}$.

**Figure 17:**
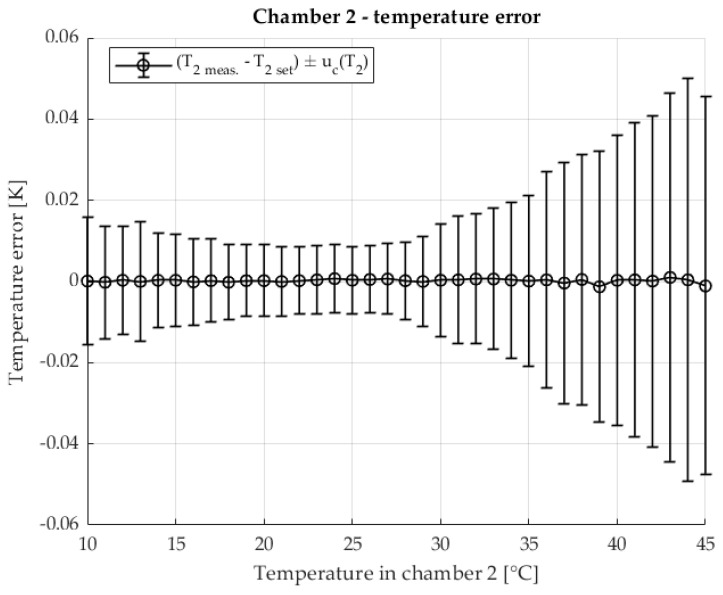
Temperature error of $T_{2}$ in chamber 2 in the two-chamber method. Resistance ratio *r* = 0.1 Ω/Ω.

Figure 17 shows the temperature stability of chamber 2, in which the test object is located. The measured temperature is displayed as a deviation from the target value, including the corresponding measurement uncertainty.

**Figure 18:**
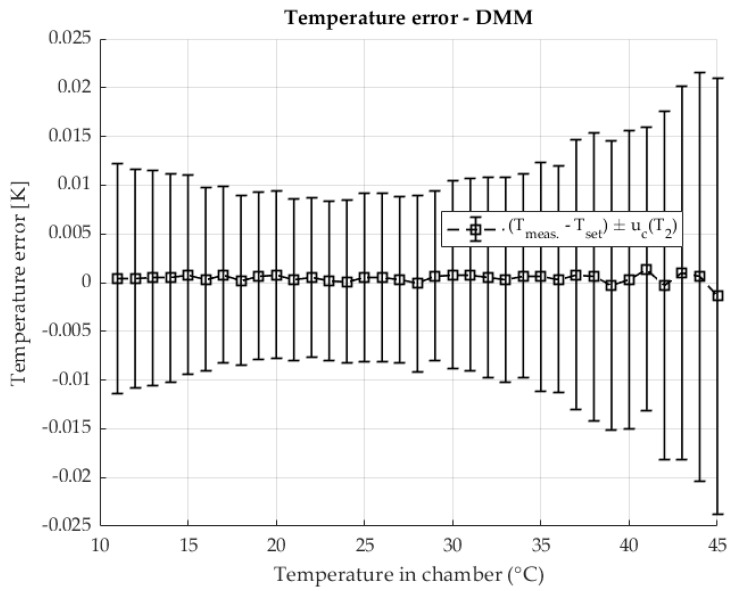
The temperature error in the chamber during the resistance measurement with the DMM. Resistance ratio *r* = 0.1 Ω/Ω

Figure 18 shows the temperature stability of resistance measurements with a DMM in four-wire mode. The results show the deviation from the target temperature, with the shaded areas indicating the uncertainty of the temperature measurement.

In this case, the temperature stability of the resistance ratio was at the same level as in previous measurements. No significant fluctuations were observed, which confirms the repeatability of the control with the described system.

## 4. Discussion

This second article (Part II) successfully focuses on the experimental implementation and validation of the novel two-chamber method, building on the theoretical framework and uncertainty analysis from Part I. The experimental results, derived from measurements over resistance ratios r of 0.1, 1 and 10, confirm the theoretical predictions from Part I. The experimental data show the robust performance of the two-chamber method, especially when compared to the classical four-wire resistance measurement with a digital multimeter (DMM).

At a low resistance ratio, the resistance of the device under test ($R_{2}$ nominal 10 Ω) optimally matches the 10 Ω measurement range of the DMM. Consequently, the DMM provides its maximum precision. The results showed good agreement between the two-chamber method and the classical DMM method, confirming that the new method is as effective as direct measurement when the resistance is optimal for the DMM’s measurement range.

When $R_{2}$ = 1 Ω, both methods generally agree, but the two-chamber method gave a smoother curve for the calculated resistance $R_{2}$ with a significantly lower measurement uncertainty. The classical DMM measurement showed fluctuations in the calculated relative TCRs around the reference temperature, emphasizing the greater consistency of the proposed method.

The last analyzed ratio, where $R_{2}$ = 0.1 Ω, was the decisive test to prove the superiority of the two-chamber approach. The small value of $R_{2}$ led to extremely unfavorable results for the classic DMM measurement. As the measured resistance was a hundred times smaller than the 10 Ω range of the DMM, the measurement range of the device was very poorly utilized, leading to a drastic increase in measurement uncertainty and the calculation of a TCR of very poor quality. In contrast, the two-chamber method remained consistent and successfully determined a high-quality TCR even with this difficult ratio.

This confirms the fundamental advantage analyzed in Part I: the new method determines the resistance $R_{t}$ indirectly via the voltage ratio $R_{t}=R_{1}(U_{t}/U_{1})$. By using a stable reference shunt $R_{1}$, which is kept at a constant temperature of 23 °C in the first chamber, the method ensures a stable and accurate determination of the current *I* for the calculation of $R_{t}$. This method effectively decouples the determination of $R_{t}$ from the measurement uncertainties caused by using the measuring range of the DMM for direct resistance measurement, especially for shunts with low resistance.

The experimental setup, including the custom relay switching unit and synchronized high-resolution DMMs (Agilent 3458A), proved critical in minimizing systematic errors. The complete four-step measurement sequence—which includes both voltmeter and resistor replacement and current polarity reversal—enabled the averaging of results, effectively canceling out systematic effects such as thermal electromotive forces, device misalignment and channel-specific errors.

The temperature control system, using a signal fusion approach (high-pass-filtered thermocouples and low-pass-filtered HygroVUE5 probes) and a digital PID algorithm, provided very stable temperature conditions in both chambers. While temperature stability was high, an increase in Type A measurement uncertainty was observed at the temperature extremes around 10 °C and 45 °C due to the increased influence of laboratory disturbances and the heating/cooling system operating at its limits. However, the analysis in Part I has shown that the uncertainty of the temperature measurements (even up to a few hundred mK) does not significantly increase the overall uncertainty budget of the TCR.

The two-chamber method represents a robust alternative to the classical methods. The system showed a high repeatability for all measured conditions. The ability of the method to maintain high precision even at unfavorable resistance ratios (low shunt resistance) means that it is ideally suited for laboratory and interlaboratory testing over a wide range of current shunts.

## 5. Conclusions

In this article (Part II), the experimental setup and validation of the novel two-chamber method for high-precision TCR determination of current shunts were successfully presented.

The experimental measurements empirically validate the theoretical framework and uncertainty analysis presented in Part I. The two-chamber approach offers a significant reduction in measurement uncertainty compared to the classical DMM four-wire resistance measurement, especially in cases with unfavorable resistance ratios. The method maintains high-quality TCR results across all tested ratios from 0.1 to 10. The increased accuracy is achieved by keeping the reference shunt $R_{1}$ at a stable temperature of 23 °C in an independently controlled chamber. In this way, the resistance of the component under test $R_{t}$ can be determined via a highly accurate voltage ratio, mitigating errors associated with the use of the DMM’s measurement range and stabilizing the determination of the reference current. The measurement setup, which uses synchronized DMMs and a four-stage sequence with polarity reversal and channel switching, effectively eliminates systematic errors. The method exhibits high repeatability, ease of use and stability, making it a robust and suitable alternative for determining TCR over a wide range of current shunts and for circular comparisons.

The established high-precision two-chamber method is used to accurately determine the TCR of new foil and cage AC shunts currently being produced in our department. These activities are related to the project “Development and validation of prototypes of innovative AC/DC shunts”, which is funded by the Recovery and Resilience Facility of the European Union.

## Figures and Tables

**Table 1:** Parameters of the resistance ratio analysis.

Parameter	Value	Unit	Distribution ^1^/ Comment
$a_{DMM}{\left(V_{DC}\right)}_{rdg}$	0.3	$\frac{\mu V}{V}$	rectangular
$a_{DMM}{\left(V_{DC}\right)}_{rng}$	0.3	$\frac{\mu V}{V}$	rectangular
DMM voltage range	1	V	DC
$a_{DMM}{\left(R\right)}_{rdg}$	5	$\frac{\mu \Omega }{\Omega }$	rectangular
$a_{DMM}{\left(R\right)}_{rng}$	3	$\frac{\mu \Omega }{\Omega }$	rectangular
DMM resistance range	10	Ω	
$R_{1}$	1	Ω	nominal @ 23 °C
$R_{0}$	(0.1, 1, 10)	Ω	nominal @ 23 °C
$I_{N}$	(10, 1, 0.1)	A	
I	Maximal allowed $I_{N}$	A	
$U{\left(R_{1}\right)}_{r}$ ^1^	2	$\frac{\mu \Omega }{\Omega }$	normal distribution
$k_{u\left(R_{1}\right)}$	1	-	coverage factor
Repeatability ^2^ of temp. sensor	40	mK	Rectangular
Long term drift of temp. sensor	30	mK/year	Rectangular

^1^ expanded calibration uncertainty of reference shunt, relative. ^2^ values are 3 standard deviations of 25 measurements at constant temperature.

## Data Availability

The inquiries can be directed to the corresponding author(s).
